# A new cable-tie based sternal closure system: description of the device, technique of implantation and first clinical evaluation

**DOI:** 10.1186/1749-8090-7-59

**Published:** 2012-06-25

**Authors:** Martin TR Grapow, Ludovic F Melly, Friedrich S Eckstein, Oliver T Reuthebuch

**Affiliations:** 1 Department of Cardiac Surgery, University Hospital Basel, Spitalstrasse 21, CH-4031, Basel, Switzerland

**Keywords:** Cable-tie, PEEK, Sternal closure

## Abstract

**Background:**

Wire closure still remains the preferred technique despite reasonable disadvantages. Associated complications, such as infection and sternal instability, cause time- and cost-consuming therapies. We present a new tool for sternal closure with its first clinical experience and results.

**Methods:**

The sternal ZipFix^TM^ System is based on the cable-tie principle. It primarily consists of biocompatible Poly-Ether-Ether-Ketone implants and is predominantly used peristernally through the intercostal space. The system provides a large implant-to-bone contact for better force distribution and for avoiding bone cut through.

**Results:**

50 patients were closed with the ZipFix^TM^ system. No sternal instability was observed at 30 days. Two patients developed a mediastinitis that necessitated the removal of the device; however, the ZipFix^TM^ were intact and the sternum remained stable.

**Conclusions:**

In our initial evaluation, the short-term results have shown that the sternal ZipFix^TM^ can be used safely and effectively. It is fast, easy to use and serves as a potential alternative for traditional wire closure.

## Background

Closure of median sternotomy with wires has been for more than 50 years and still is the gold-standard worldwide. The technique is easy, fast, safe, reproducible and cheap. However, the patient population undergoing cardiac surgery today has dramatically changed. With the introduction of interventional cardiology in the late seventies and the significantly improved techniques in cardiac surgery, anaesthesia and intensive care medicine, the surgical candidate has shifted from a more or less healthy patient with a cardiac problem to a multimorbid one with a serious cardiac disease. Advanced age, diabetes, obesity, renal failure, lung diseases, osteoporosis, poor nutritional status as predisposing patient-related factors on one hand are compounded by more complex operations on the other hand.

The above listed comorbidities have crucial impact on bone architecture, composition of corticalis and spongiosa. Furthermore, they significantly influence bone and wound healing. Wires may be the perfect closing strategy in a strong and durable sternum, but might be suboptimal in a weak and soft bone. With the first powerful cough, the wire can tear through the sternum. The force is concentrated on a very tiny surface length of the wire, which then can act like a knife. The consequences are, at least, uncomfortable reoperations, but can of course be worsen from accompanying infection up to life-threatening mediastinitis.

Many different devices have been developed to overcome this problem during the past two decades, but most of them failed because of a mismatch in practicability and economy. This article presents a very promising technique that is new for sternal closure, but is otherwise well-known in various fields – the cable tie principle, which is incorporated in the Synthes ZipFix^TM^–implant (Synthes GmbH, Oberdorf, Switzerland). The device has been designed to be best applicable for sternal closure.

### Technology

**Figure 1:**
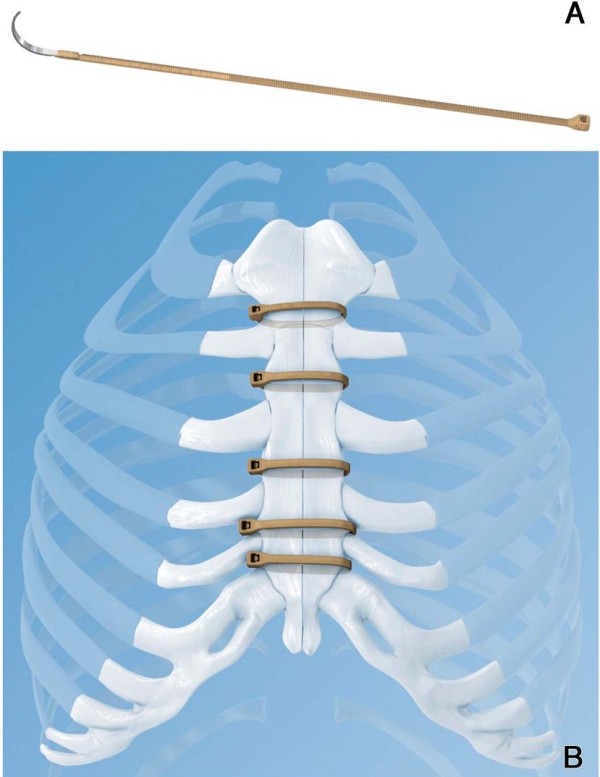
(**A**) **ZipFix**^**TM**^**System with the blunt stainless steel needle.** (**B) 5 implants applied as recommended by the manufacturer.**

The Sternal ZipFix^TM^ System is made of PEEK (Poly-Ether-Ether-Ketone) with an attached blunt stainless steel needle. Typically 5 implants are recommended per midline sternotomy closure (Figure 1). After the removal of the needle the end is inserted into the locking head. The tensioning of each ZipFix^TM^ is performed with its system specific application instrument, that limits the maximum tension applied to the device to prevent over-tensioning and its damaging.

The relatively flexible and wide sternal ZipFix^TM^ device provides a large implant-to-bone contact area with a width of 4.2 mm (versus 0.7 mm for USP 5 steel wire), reducing the risk of cut through of the sternal bone. It also provides a low profile height over the sternum bone and can be used in patients with Nickel allergies. The material PEEK is MRI safe and not visible in a standard x-ray image. The biocompatibility has been first confirmed in the late 1980s by Williams et al. [1]. By the late 1990s PEEK emerged as the leading high-performance thermoplastic material for replacing some metal implant devices and showed excellent resistance in simulated “in vivo” degradation, including damage caused by lipid exposure [2].

**Figure 2:**
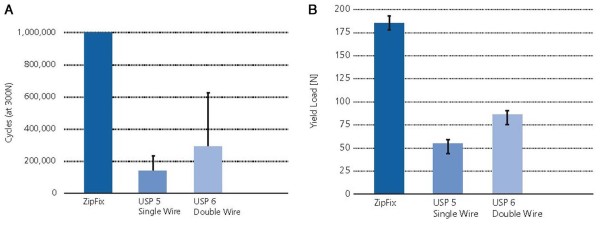
(**A**) **Fatigue test with exaggerated, dynamic loading intended to mimic physiologic conditions and** (**B**) **simulation of bone cut-through of three different implants.**

The mechanical function of the system was characterized by the manufacturer with assessment of strength and durability as compared to USP 5 stainless steel surgical wire for sternal closure. Each device was prepared in test fixtures to simulate peristernal application and subjected to lateral loading. Both were first loaded statically in tension until failure to determine their strength. The yield load for ZipFix™ was 425 ±18.1 N and 299 ±11.5 N for the USP 5 stainless steel wire. Additional implants were loaded in the same manner and subjected to exaggerated, dynamic loading intended to mimic physiologic conditions for up to one million cycles - representing over six weeks of respiration. At a max load of 300 N, the ZipFix™ survived one million cycles; whereas, the USP 5 stainless steel wire survived 148,041 ±114,394 cycles (Figure 2A).

To assess the implant cutting through the bone as a failure mode, tests were performed in 12 mm thick polyurethane foam blocks of 10 lb/ft^3^, simulating poor-quality bone. Failure occurred at loads of 184 ±7.0 N and 52 ±5.3 N, for ZipFix™ and USP 5 stainless steel wire respectively. To summarize the mechanical testing shows that ZipFix™ has at least equivalent or better performance compared to stainless steel surgical wire with respect to static loading strength, fatigue strength, and resistance to cut-through (Figure 2B).

### Surgical technique

#### Technique #1

**Figure 3:**
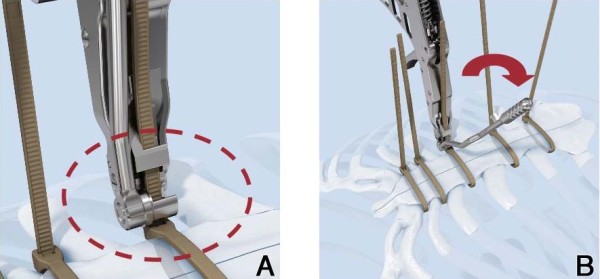
**The application device** (**A**) **closes each ZipFix**^**TM**^**with a force of 200 N and** (**B**) **excess material is cut on the level of the lock.**

**Figure 4:**
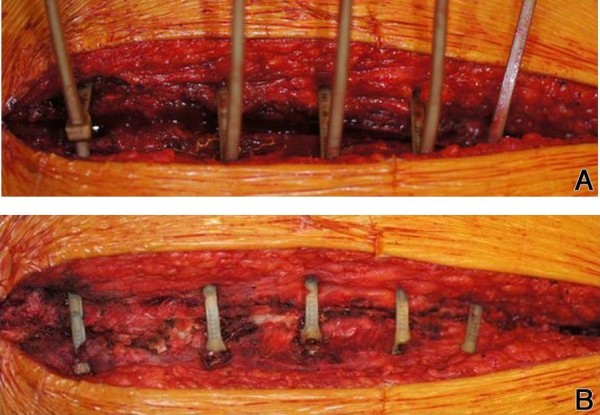
**Intraoperative view** (**A**) **with all five ZipFix**^**TM**^**after hand-tightening and** (**B**) **final result.**

When haemostasis is finished, tubes placed, and the pericardium closed, the first ZipFix^TM^ is placed through the manubrial bone with at least 1 cm distance to the sternotomy on both sides, this may be difficult or impossible by thick manubrium or strong corticalis (See Technique #2) . The next three ZipFix^TM^ are placed directly surrounding the sternum through the intercostal spaces. The fifth ZipFix^TM^ often has to be passed through the bone again, which is easier due to a weaker bone-cartilage composition in the xyphoidal region. The needles are cut after each placement. The blind ends are passed through the lock and all five ZipFix^TM^ are hand-tightened. Attention has to be paid for approximating both lateral borders on the same level in order to avoid any step. With the application device each ZipFix^TM^ is closed with a force of 200 N (Figure 3A) and excess material cut on the lock level (Figure 3B), which remains in the right intercostal spaces. In (cachectic) patients with little subcutaneous tissue, placing the ZipFix^TM^ through the bone precludes the ability to countersink the lock onto the bone level, otherwise it may lead to a cosmetically suboptimal and painful result (Figure 4).

#### Technique #2

In case of strong manubrial bone or cachectic patient (prominent lock), as an alternative a wire can be used cranially or caudally instead of the ZipFix^TM^. In the middle part of the sternum the three ZipFix^TM^ can be applied in the intercostal spaces as described in technique #1.

## Methods

Data were collected, reviewed and analyzed retrospectively. Study protocol was approved by the ethical committee of the University of Basel. 50 elective patients, who underwent cardiac surgery via median sternotomy, were closed with the ZipFix^TM^ system. Euroscore was applied to assess patients` perioperative risk. Patient demographics are depicted in Table 1. Almost half of the patients (24/50) had combined procedures and seven patients underwent emergency operations. Closure was performed by two senior consultants. At the time of surgery, the type of sternal closure was designated according to cumulative preoperative risk factors as well as sternal quality, e.g. sternal width, sternal height and consistence.

**Table 1:** Patient Demographics

	**Range**	**Mean**
**Sex**	22 females/28 males	
**Age**	[35–81]	67 ± 12
**BMI** [kg/m^2^]	[19–44]	27 ± 6
**Diabetes**		*21/50*
**Pulmonary Disease/Smoker**		*36/50*
**Operative Time** [min]	[90–330]	200 ± 50
**Left ventricular ejection fraction** [%]	[15–80]	51 ± 14
**Logistic Euroscore** [%]	[1–84]	15 ± 19

Mean value ± standard deviation.

## Clinical experience and Results

Thus 37/50 were closed with ZipFix^TM^ solely and 13/50 were stabilized in conjunction with conventional wires (Fumedica, Reichshof, Germany). A total of 4.7 ± 0.7 ZipFix^TM^ were implanted in each patient. We observed a gradual reduction in the mean time of implantation from 15 minutes for the first cases, which corresponds to sternal closure with wires, to 7 minutes for the last 20 cases. There was no bleeding due to lesions of intercostal or the remaining internal mammary arteries. One patient died at day 14 due to septic shock of abdominal origin, unrelated to the sternal closure. Clinical examination confirmed sternal stability in all patients at discharge from the hospital and in 47/49 at 30 days postoperatively. Indeed, 2 patients developed mediastinitis that necessitated removal of the ZipFix^TM^ at day 24 and 30 in association with administration of antibiotics. At that time, both sterna were stable and all ZipFix^TM^ were intact. Both patients were suffering from metabolic syndrome with a BMI of 40 and 35 respectively, as well as insulin-dependent diabetes and chronic renal failure under hemodialysis. Postoperatively, 2 female patients had to be mechanically resuscitated. In the first case at day 3 the patient received 2 shocks of 200 J for a ventricular fibrillation during the 10 minutes resuscitation. In the second case the patient showed an asystoly at day 19 and was reanimated during 35 minutes. Even though osteoporotic, both sterna remained stable.

## Discussion

In 1958, the AO (Arbeitsgemeinschaft für Osteosynthesefragen) formulated four basic principles, which have become the guidelines for internal fixation [3]: restoration of anatomical relationships, stable fixation, preservation of blood supply and early and safe mobilization. For more than half a century, steel wires were predominantly used to close the chest after cardiac surgery after median sternotomy. In between the profession has changed dramatically. Procedures and anesthesia are faster, less invasive, less harmful, and medication is more target-oriented. This results in a tremendous shift towards significantly older and sicker patients with comorbidities that limit survival and have crucial impact on wound and sternal healing.

Although a variety of different closure devices have been introduced in the past two decades, none has been able to fully replace wires in terms of practicability and costs. Sternal dehiscence, wound infections, mediastinitis, sternal fractures and non-unions are still unsolved problems that arise in 3-5% [4] of treated patients. Follow-up costs remain a considerable burden on health care systems [5,6].

The ZipFix^TM^ System is based on the cable-tie principle, which enables rigid fixation for use in primary sternal closure. The implantable device is exclusively made from PEEK, which has emerged as the leading high-performance thermoplastic material for many industries, including medical devices. By replacing some metal implant devices, PEEK has shown excellent stress resistance characteristics, biocompatibility and resistance in simulated “in vivo” degradation, including damage caused by lipid exposure [2].

Initial tests performed by Synthes demonstrated the superior fatigue strength of sternal ZipFix^TM^ compared with stainless steel cerclage wires. Furthermore, due to the large implant to bone contact area, which results in an optimized stress distribution, the force to achieve bone-cut-through has to be significantly higher. A similar approach has been ventured by Sterna-Band^TM^. Compared with wires, these steel bands not only provided effective fixation, they demonstrated a reduction in postoperative pain and length of postoperative hospital stay [7,8]. The advantages of the ZipFix^TM^ over the Sterna-Band^TM^ are the ease and speed of implantation and the reproducible tension of 200 N for each ZipFix^TM^. In case of an emergency, the ZipFix^TM^ are easily cut by scissors. The “soft and smooth” material adapts perfectly to the bone. Additionally, the lack of sharp edges might be less vulnerable to the periostium. Thus, all four key principles formulated by the AO are respected in this device.

Concerning the costs of this new device they are, at the moment being, about 5 to 8 times more expensive than the conventional wires and though cannot be ignored. But as all new devices, prices will tend to be reduced as the use increases. In addition if stability can be improved even in extreme situation such as mechanical reanimation, some extra cost of reoperation for sternal refixation could be avoided. This cost effectiveness must now be examined.

Having implanted the first ZipFix^TM^ worldwide, we present our short-term results in the inital 50 patients. None of them experienced sternal instability, including the two with mediastinitis and the other two female patients after successful external cardiac resuscitation. Since we decided to test this new CE certified product in our daily practice we did not conduct a randomized trial for our first clinical experience which is definitely a limitation to our study and this question has to be answered by newly conducted randomized controlled trials in the near future with special attention on health care costs.

## Conclusion

However our initial short-term results demonstrated, in a small group of patients, the safe and effective use of the sternal ZipFix^TM^ System. It is fast, reproducible, easy to use and has great potential for being an alternative for traditional wire closure.

## Competing interests

The author declare that they have no competing interests. This research received no specific grant from any funding agency in the public, commercial, or not-for-profit sectors. Sternal ZipFix^TM^ System was purchased on a regular basis. The authors had full control of the design of the study, methods used, outcome parameters and results, analysis of data and production of the written report.

## Authors’ contributions

MG performed the implantation of ZipFixTM and drafted the manuscript, LM collected, analyzed and interpreted the data, FE critically revised the manuscript and is responsible for concept and design, and OR implanted the ZipFixTM and helped drafting the manuscript. All authors read and approved the final manuscript.
